# Reply: ZD6474 reverses multidrug resistance by directly inhibiting the function of P-glycoprotein

**DOI:** 10.1038/sj.bjc.6604078

**Published:** 2007-11-06

**Authors:** Y Mi, L Lou

**Affiliations:** 1 Chinese Academy of Sciences, Shanghai Institute of Materia Medica, 555 Zuchongzhi Road, Shanghai 201203, China

**Sir**,

In the article ZD6474 reverses multidrug resistance by directly inhibiting the function of P-glycoprotein in *British Journal of Cancer* (2007) **97**: 934–940, we indeed did not check the authenticity of the cell lines MCF-7/ADR and KB, so, it is likely that MCF-7/ADR is not an authentic variant of the MCF-7 breast carcinoma cell line and that KB is not derived from oral carcinoma, although there are numerous papers to mention that MCF-7/ADR is a variant of the MCF-7 breast carcinoma cell line and KB is derived from oral carcinoma. However, the authenticity of these cell lines does not compromise the conclusion and scientific significance of this paper. In our article, it is very clear that these cell lines (MCF-7/ADR and KBV200) are drug resistant and their drug resistances are mediated by P-glycoprotein. These characteristics of these cell lines are enough to justify them to be used to study the pharmacologic effects of ZD6474 and the mechanisms involved.

